# Structural and Energetic Insights on Two Dye Compounds: 1-Acetyl-2-Naphthol and 2-Acetyl-1-Naphthol

**DOI:** 10.3390/molecules25173827

**Published:** 2020-08-22

**Authors:** Vera L. S. Freitas, Maria D. M. C. Ribeiro da Silva

**Affiliations:** Centro de Investigação em Química da Universidade do Porto (CIQUP), Department of Chemistry and Biochemistry, Faculty of Sciences, University of Porto, Rua do Campo Alegre, P-4169-007 Porto, Portugal; mdsilva@fc.up.pt

**Keywords:** *o*-hydroxyacetonaphthone isomers, tautomers, excited state intramolecular proton transfer, intramolecular hydrogen bond, combustion calorimetry, Knudsen effusion method

## Abstract

The energy involved in the structural switching of acyl and hydroxyl substituents in the title compounds was evaluated combining experimental and computational studies. Combustion calorimetry and Knudsen effusion techniques were used to determine the enthalpies of formation, in the crystalline state, and of sublimation, respectively. The gas-phase enthalpy of formation of both isomers was derived combining these two experimental data. Concerning the computational study, the G3(MP2)//B3LYP composite method was used to optimize and determine the energy of the isomers in the gaseous state. From a set of hypothetical reactions it has been possible to estimate the gas-phase enthalpy of formation of the title compounds. The good agreement between the experimental and computational gas-phase enthalpies of formation of the 1-acetyl-2-naphthol and 2-acetyl-1-naphthol isomers, provided the confidence for extending the computational study to the 2-acetyl-3-naphthol isomer. The structural rearrangement of the substituents in position 1 and 2 in the naphthalene ring and the energy of the intramolecular hydrogen bond are the factors responsible for the energetic differences exhibited by the isomers. The gas phase tautomeric keto ↔ enol equilibria of the *o*-acetylnaphthol isomers were analyzed using the Boltzmann’s distribution.

## 1. Introduction

**Figure 1:**
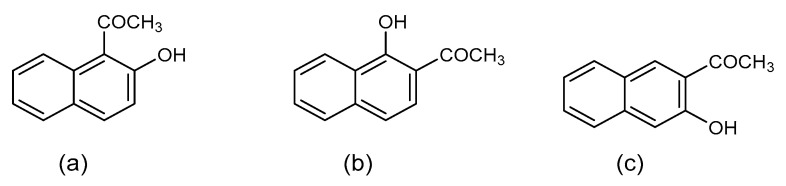
Structural formulae of 1-acetyl-2-naphthol (**a**), 2-acetyl-1-naphthol (**b**), and 2-acetyl-3-naphthol (**c**).

The *o*-acetylnaphthol isomers (structural formulae presented in Figure 1) present an orientation and close proximity of the oxygen of the acetyl group towards the hydrogen of the hydroxyl group, enabling the formation of an intramolecular hydrogen bond (IMHB) [1,2,3,4,5,6,7,8]. The formation of IMHBs in organic compounds can provide them photophysical properties with a multiplicity of applications in various scientific and technological domains. Generally, upon photo-excitation of these compounds a fast acid–base reaction can occur via the IMHB resulting in a tautomeric transformation from the ground state enol form to an excited keto form. This process is known as excited state intramolecular proton transfer (ESIPT). The most remarkable photophysical property of the ESIPT chromophores is the large fluorescence Stokes shift. The presence of this property in compounds showing ESIPT phenomena, together with the dual emission makes them quite exploited as OLED materials, spectroscopy tools, and fluorescence probes in biology [9,10,11,12,13]. However, the ESIPT process in 2-acetyl-1-naphthol has been in controversy for long, primarily because of its quantitative ESIPT conversion in most solution conditions and also due to small Stokes shift of the ESIPT products (keto type prototautomer) compared to those products in other typical ESIPT molecules [1,14]. This doubtfulness remained until Kim et al. [15] had performed time-resolved fluorescence studies of 2-acetyl-1-naphthol in solution phase involving measurements with high time resolution, confirming the ESIPT phenomena in the compound. Very recently, Ning et al. [16], has further re-established the presence of ESIPT process in 2-acetyl-1-naphthol, estimating it to take place in the time scale of about 37 fs and occurring with a small energy barrier of about 1.0 kJ⋅mol^−1^ in the S_1_ state of the dye.

This work follows a previous thermochemical study involving other remarkable ESIPT chromophore, the 2-hydroxy-1-naphthaldehyde [17].

The static bomb combustion calorimetry was used on the measurement of the energy of combustion of 1-acetyl-2-naphthol and 2-acetyl-1-naphthol, which allowed to derive the corresponding standard (*p* = 0.1 MPa) molar enthalpies of formation, in the crystalline phase, at *T* = 298.15 K. The vapor pressures of the two isomers were measured, at several temperatures, by Knudsen effusion technique, and by application of the Clausius–Clapeyron equation to the results obtained, the molar enthalpies and entropies of sublimation were derived. The values of the standard molar enthalpy of formation, in the crystalline phase, and the standard molar enthalpy of sublimation were combined to derive the standard molar enthalpy of formation, in the gaseous phase, of the title compounds at *T* = 298.15 K. The values of the gaseous enthalpy of formation of these compounds were also derived from G3(MP2)//B3LYP calculations [18] considering a conformational equilibrium composition of the molecular structures and the energy of several hypothetical group substitution reactions. Experimental and computational results were compared and discussed. The computational study was also extended to isomer 2-acetyl-3-naphthol. The energetics of the intramolecular hydrogen bond (O⋅⋅⋅H) present in *o*-acetylnaphthol isomers were investigated using theoretical methods. Additionally, the gas phase tautomeric keto ↔ enol equilibria of the *o*-acetylnaphthol isomers were also analyzed using the Boltzmann’s distribution.

## 2. Results

### 2.1. Experimental Studies

#### 2.1.1. Combustion Calorimetry—Energy of Combustion

Static-bomb combustion calorimetry of the isoperibol type was the technique used to perform the combustion experiments in oxygen for the two *o*-acetylnaphthol isomers [19]. Six combustion experiments were carried out for each dye compound, whose results are reported in Tables S1 and S2 given in Supplementary Materials. All values in those tables refer to the combustion reaction described in Equation (1).

(1) $$C_{12}H_{10}O_{2}(\mathrm{cr})\;+\;13{.5\;O}_{2}(g)\;\to {\;12\;\mathrm{CO}}_{2}{(g)\;+\;5\;H}_{2}O$$

The energy associated to the isothermal bomb process, Δ*U*(IBP), was calculated through Equation (2), where *ε*(cal) is the energy equivalent of the calorimeter (obtained from calibration with certified benzoic acid), Δ*m*(H_2_O) represents the deviation of the mass of water added to the calorimeter from 2900.0 g (the reference quantity of water assigned to the energy equivalent *ε*(cal)), *c_p_*(H_2_O, l) is the massic heat capacity at constant pressure for liquid water, *ε*_f_ is the energy equivalent of the contents in the final state, Δ*T*_ad_ is the calorimeter temperature corrected for the heat exchange, and work of stirring, and Δ*U*(ign) is the electric energy for the ignition.

(2) $$\Delta U\left(\mathrm{IBP}\right)=-\{\varepsilon \left(\mathrm{cal}\right)+\;\Delta m\left(H_{2}O\right)\cdot c_{p}\left(H_{2}O,\;l\right){+\;\varepsilon }_{f}\}\Delta T_{\mathrm{ad}}+\;\Delta U\left(\mathrm{ign}\right)$$

The standard state corrections, designated Washburn corrections, Δ*U*_Σ_, were calculated as recommended in the literature for organic compounds [20].

The standard molar energies, $\Delta_{c}U_{m}^{{}^{\circ}}\left(\mathrm{cr}\right),$ and enthalpies, $\Delta_{c}H_{m}^{{}^{\circ}}\left(\mathrm{cr}\right)$, of combustion reaction described by Equation (1) for each isomer are reported in Table 1. The standard molar enthalpies of formation, $\Delta_{f}H_{m}^{{}^{\circ}}\left(\mathrm{cr}\right)$, in the crystal phase are also reported in the same table, calculated from the corresponding $\Delta_{c}H_{m}^{{}^{\circ}}\left(\mathrm{cr}\right)$, and the standard molar enthalpies of formation of H_2_O(l), CO_2_(g), at *T* = 298.15 K, respectively, (−285.830 ± 0.042) kJ·mol^−1^ [21] and (−393.51 ± 0.13) kJ·mol^−1^ [21]

#### 2.1.2. Knudsen Mass-Loss Effusion Method—Enthalpy of Sublimation

The Knudsen mass-loss effusion method was used to determine the sublimation vapor pressures, at different temperatures, of the two *o*-acetylnaphthol isomers. The measurements were performed in the temperature region chosen to correspond to vapor pressures between 0.1 and 1.0 Pa: *T* = (306 and 328) K for 1-acetyl-2-naphthol and *T* = (312 to 336) K for 2-acetyl-1-naphthol.

The Knudsen system used allows the simultaneous operation of nine effusion cells, arranged in three independent ovens (maintained at a given controllable temperature), each of them contains three cells with different sized effusion orifices (small, medium, and large). In this manner, by keeping the same temperature for each group of three effusion cells with different orifice areas, deviation of results from the equilibrium pressures may be checked [23].

The saturation vapor pressure, *p*_i_**,** of the effusing compound *i* can be formulated using the kinetic theory of gases, according expression given by Equation (3), where $A_{0}$ is the area of the orifice (given by $\pi r_{0}^{2}$, where $r_{0}$ is the radius of the orifice, with typical orifice areas on the order of 10^−6^ m^2^), $\omega_{0}$ is the transmission probability Clausing factor, *T* is temperature (oven temperature where the cell is placed), $m_{i}$ is the mass of the sample sublimed from the effusion cell, *t* is the period time of the experiment, $M_{i}$ is the molar mass of the effusing vapor, and *R* is the gas constant (*R* = 8.314462618 J⋅K^−1^⋅mol^−1^) [24].

(3) $$p_{i}=\;\frac{m_{i}}{A_{0}\omega_{0}t}\sqrt{\frac{2\pi \mathrm{RT}}{M_{i}}}$$

The results of the vapor pressure, at the different temperatures, obtained for the two *o*-acetylnaphthol isomers, together with all the necessary parameters for its calculation are given in Supplementary Materials in Table S3.

For crystal–vapor equilibrium, the Clausius–Clapeyron equation gives the quantitative dependence of the vapor pressure of the crystal on temperature. This equation is expressed in Equation (4) where, *p* is the vapor pressure, *T* is the temperature, $\Delta_{\mathrm{cr}}^{g}H_{m}^{{}^{\circ}}$ is the standard molar enthalpy of sublimation, and *R* is the gas constant [24]. For temperature interval lower than 20 K, the enthalpy of sublimation can be considered to be approximately constant (regardless of temperature, $d\left(\Delta_{\mathrm{cr}}^{g}H_{m}^{{}^{\circ}}\right)/dT=0$).

(4) $$\frac{\mathrm{dln}p}{dT}=\;\frac{\Delta_{\mathrm{cr}}^{g}H_{m}^{{}^{\circ}}}{{\mathrm{RT}}^{2}}$$

**Figure 2:**
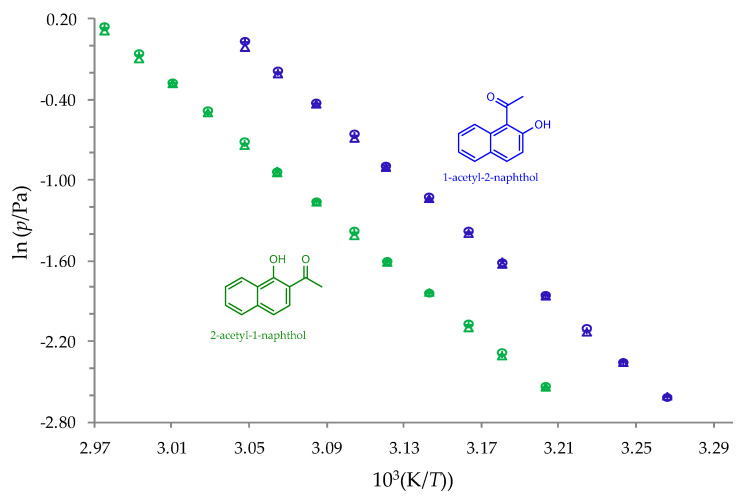
Plots of ln(*p*/Pa) against 1/*T* for small “o”, medium “+” and large “Δ” orifices obtained by the mass-loss Knudsen effusion experiments for 1-acetyl-2-naphthol (marked with blue colcor) and 2-acetyl-1-naphthol (marked with green color).

From the integrated form of Clausius–Clapeyron equation (Equation (4)) a plot of ln*p* versus 1/*T* is a straight line with slope $-\Delta_{\mathrm{cr}}^{g}H_{m}^{{}^{\circ}}/R$. The graphical representations of ln *p* = *f*(1/*T*) for small, medium and large orifices obtained for 1-acetyl-2-naphthol and 2-acetyl-1-naphthol are illustrated in Figure 2.

The Clausius-Clapeyron equation were represented as follows ln(p/Pa)=a−b(K/T), where the parameters *a* is a constant and $b=\;\Delta_{\mathrm{cr}}^{g}H_{m}^{{}^{\circ}}\left(\langle T\rangle \right)/R$. The results of the parameters of the Clausius-Clapeyron equation obtained from the least square adjustment for each set of effusion cells (small, S; medium, M; and large, L) and for the global treatment of all the results obtained for 1-acetyl-2-naphthol and 2-acetyl-1-naphthol, are in given Table S4 of Supplementary Materials. The good agreement between the parameters obtained for each set of orifices of each compound leads to the conclusion that there are no deviations from the results of the equilibrium pressures for both isomers. In light of this, the global results will be used in the calculation of the sublimation thermodynamic properties at reference temperature. At the same table are also reported the equilibrium pressure at the mean temperature, (*p*〈*T*〉), together with the standard molar enthalpies of sublimation at the mean temperature of the experiments, $\Delta_{\mathrm{cr}}^{g}H_{m}^{{}^{\circ}}\left(\langle T\rangle \right)$, and the standard molar entropy of sublimation at the equilibrium pressure and mean temperature, $\Delta_{\mathrm{cr}}^{g}S_{m}^{{}^{\circ}}\left(\langle T\rangle ,p\langle T\rangle \right)$, calculated by Equation (5).

(5) $$\Delta_{\mathrm{cr}}^{g}S_{m}\left(\langle T\rangle ,\;p\left(\langle T\rangle \right)\right)\;=\;\Delta_{\mathrm{cr}}^{g}H_{m}^{{}^{\circ}}\left(\langle T\rangle \right)/\langle T\rangle$$

The values of the derived standard molar enthalpy, $\Delta_{\mathrm{cr}}^{g}H_{m}^{{}^{\circ}}$, entropy, $\Delta_{\mathrm{cr}}^{g}S_{m}^{{}^{\circ}}$, and Gibbs energy of sublimation, $\Delta_{\mathrm{cr}}^{g}G_{m}^{{}^{\circ}}$, at *T* = 298.15 K, together with the vapor pressure at the same temperature, are reported in Table 2. These parameters were calculated using Equations (6)–(8), respectively. The difference in standard molar heat capacity change at constant pressure between the gaseous and the crystalline phase used, $\Delta_{\mathrm{cr}}^{g}C_{p,m}^{{}^{\circ}}$, was calculated through Equation (9) derived by Monte et al. [25] as a rearrangement of Equation (10) suggested by Chickos et al. [26], using the value of the standard molar heat capacity at constant pressure in the gaseous phase at *T* = 298.15 K, $C_{p,m}^{{}^{\circ}}\left(g\right)$. Statistical thermodynamics via the vibrational frequencies, calculated at the B3LYP/6-31G(*d*) level of theory (scaled by a factor of 0.960 ± 0.022) [27], were used to calculate the $C_{p,m}^{{}^{\circ}}\left(g\right)$ of the *o*-acetylnaphthol isomers. The values of $C_{p,m}^{{}^{\circ}}\left(g\right)$ used and the $\Delta_{\mathrm{cr}}^{g}C_{p,m}^{{}^{\circ}}$ obtained were, respectively: 200.34 J⋅K^−1^⋅mol^−1^ and −36.16 J⋅K^−1^⋅mol^−1^ for 1-acetyl-2-naphthol; 201.06 J⋅K^−1^⋅mol^−1^ and −36.29 J⋅K^−1^⋅mol^−1^ for 2-acetyl-1-naphthol.

(6) $$\Delta_{\mathrm{cr}}^{g}H_{m}^{{}^{\circ}}\left(298.15K\right)/\mathrm{kJ}\cdot {\mathrm{mol}}^{-1}=\;\Delta_{\mathrm{cr}}^{g}H_{m}^{{}^{\circ}}\left(\langle T\rangle \right)-\;\Delta_{\mathrm{cr}}^{g}C_{p,m}^{{}^{\circ}}\left(\langle T\rangle -298.15\right)$$

(7) $$\Delta_{\mathrm{cr}}^{g}S_{m}^{{}^{\circ}}\left(298.15K\right)/J\cdot K^{-1}\cdot {\mathrm{mol}}^{-1}=\;\Delta_{\mathrm{cr}}^{g}S_{m}^{{}^{\circ}}\left(\langle T\rangle ,p\langle T\rangle \right)+R\ln\left[p\left(\langle T\rangle \right)/p^{{}^{\circ}}\right]+\;\Delta_{\mathrm{cr}}^{g}C_{p,m}^{{}^{\circ}}\ln\left(298.15/\langle T\rangle \right)$$

(8) $$\Delta_{\mathrm{cr}}^{g}G_{m}^{{}^{\circ}}\left(298.15K\right)/\;\mathrm{kJ}\cdot {\mathrm{mol}}^{-1}=\;\Delta_{\mathrm{cr}}^{g}H_{m}^{{}^{\circ}}\left(298.15\right)-\;298.15\cdot \Delta_{\mathrm{cr}}^{g}S_{m}^{{}^{\circ}}\left(298.15\right)$$

(9) $$\Delta_{\mathrm{cr}}^{g}C_{p,m}^{{}^{\circ}}/J\cdot K^{-1}\cdot {\mathrm{mol}}^{-1}\;=\;-\left\{0.9+0.176\;C_{p,m}^{{}^{\circ}}\left(g\right)\right\}$$

(10) $$\Delta_{\mathrm{cr}}^{g}C_{p,m}^{{}^{\circ}}/J\cdot K^{-1}\cdot {\mathrm{mol}}^{-1}\;=\;-\left\{0.75+0.15\;C_{p,m}^{{}^{\circ}}\left(g\right)\right\}$$

### 2.2. Computational Studies

The computational study of the three *o*-acetylnaphthol isomers (Figure 1) was performed using the G3(MP2)//B3LYP approach [18]. The good agreement obtained between the experimental and the computational values for the gas-phase enthalpy of formation in previous studies of compounds with a similar structural typology, give us confidence for the use of the G3(MP2)//B3LYP approach in this study [17,28,29,30].

**Figure 3:**
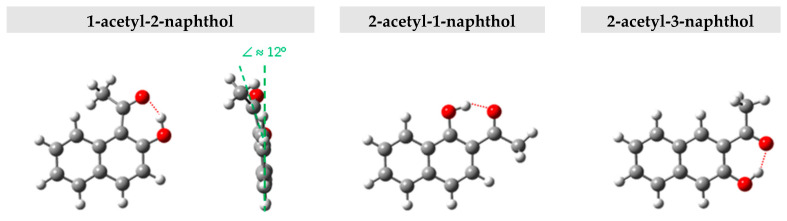
Optimized geometries corresponding to the global minima of the *o*-acetylnaphthol isomers obtained by the G3(MP2)//B3LYP composite method. Atom color code: grey, C; red, O; white, H.

Due to the different possible structural arrangements of the acyl and hydroxyl substituents in the gaseous phase, it was obtained several minima in the potential energy surface. The conformational composition, χ_i_, for each *o*-acetylnaphthol isomer was calculated assuming a Boltzmann distribution of the *n* possible equilibrium structures, using for that the estimated entropy of formation, $\Delta_{f}S_{m}^{{}^{\circ}}\left(g\right)$, and Gibbs energy of formation, $\Delta_{f}G_{m}^{{}^{\circ}}\left(g\right)$, in the gaseous phase. Details of the conformational analysis performed are given in Supplementary Materials in Tables S5–S7. The global minimum conformer (with lowest gas-phase Gibbs energy of formation, with a χ_i_ equal to 1), corresponds to the structure where the IMHB occurs. In Figure 3 the optimized geometries are presented corresponding to the global minimum conformer (lowest $\Delta_{f}G_{m}^{{}^{\circ}}\left(g\right)$) obtained for each *o*-acetylnaphthol isomer. The structures of 2-acetyl-1-naphthol and 2-acetyl-3-naphthol are coplanar, while the same is not true for isomer 1-acetyl-2-naphthol, where the acetyl group is slightly distorted about 12° outside the plane of the rings.

The presence of the IMHB in the gas-phase were confirmed by the topological parameters of the electron density at the selected bond critical point obtained by quantum theory of atoms in molecules (AIM) [31,32]. The IMHB length in the gaseous phase and the electron density at the bond critical point, *ρ*_BCP_, obtained by quantum theory of atoms in molecules (AIM) are reported in Table 3, together with the energy of the intramolecular hydrogen bond, $E_{\mathrm{IMHB}}$, calculated by conformational analysis and by the *ortho-para* method. The $E_{\mathrm{IMHB}}$ by the conformational analysis was calculated from the difference of the ground state structure (global minimum), stabilized by the IMHB, and the second most stable conformer (local minimum) where the IMHB is not present (the values and the molecular structures of the conformers are given in Supplementary Materials in Tables S5–S7). According with the *ortho-para* method proposed by Estácio et al. [33], the $E_{\mathrm{IMHB}}$ is calculated from the difference of the energies of the most stable *ortho* and *para* isomers, at *T* = 298.15 K. The *para* isomer used was the 1-acetyl-4-naphthol. The conformers used in this calculation correspond to the global minimum in the potential energy surface obtained by the G3(MP2)//B3LYP method. The IMHB values of the 2-acetyl-1-naphthol and 2-acetyl-3-naphthol isomers obtained by the two methods are consistent; the same is not true for the 1-acetyl-2-naphthol isomer. A possible explanation for this difference is the non-cancellation of the structural distortion caused by the acetyl group in the isomer 1-acetyl-2-naphthol, when it is used the *ortho*-*para* method, since the isomer *para*, the 1-acetyl-4-naphthol, it is completely planar. While, in the conformational analysis this structural distortion is also present in the open ring isomer (second most stable conformer), which enables the cancelling of this effect.

The substitution group reactions used in the estimation of the gas-phase enthalpy of formation of *o*-acetylnaphthol isomers, like isodesmic and homodesmotic reactions, relies upon the similarity of the bonding environment of the reactants and product and lead to cancellation of systematic errors. These type reactions have the disadvantage of requiring experimental gas-phase enthalpies of formation of all auxiliary molecules used. To avoid the possible use of any inaccurate experimental enthalpy of a given species, 16 working reactions were used. For each working reaction, the value of the gas-phase enthalpy of formation of the compound studied was calculated from the enthalpy at *T* = 298.15 K, $H_{298.15K}^{{}^{\circ}}$, obtained computationally from the G3(MP2)//B3LYP method [18] and the experimental standard molar gas-phase enthalpies of formation of all the auxiliary species used [17,34,35,36,37,38,39] (all the values are given in Supplementary Materials in Table S8). The purposed working reactions and the corresponding enthalpies of formation calculated for each isomer are given in Table S9.

The gas phase tautomeric keto ↔ enol equilibria of the *o*-acetylnaphthol isomers were also analyzed using the Boltzmann’s distribution, with results reported in Supplementary Materials in Table S10. For the three *o*-acetylnaphthol isomer the fraction *x*_enol_ is equal to 1, thus leading us to conclude that enol form is the most stable in the gas phase.

## 3. Discussion

The overall thermodynamic results obtained for *o*-acetylnaphthol isomers throughout this work are compiled in Table 4.

The experimental values of the $\Delta_{f}H_{m}^{{}^{\circ}}\left(g\right)$ for 1-acetyl-2-naphthol and 2-acetyl-1-naphthol were calculated through Equation (11), combining the $\Delta_{f}H_{m}^{{}^{\circ}}\left(\mathrm{cr}\right)$ obtained by combustion calorimetry and the $\Delta_{\mathrm{cr}}^{g}H_{m}^{{}^{\circ}}$ by Knudsen effusion method. This gas-phase thermodynamic parameter was also estimated using theoretical calculations. The excellent agreement between the experimental and computational $\Delta_{f}H_{m}^{{}^{\circ}}\left(g\right)$ values, considering the inherent uncertainty, gave us confidence to estimate the $\Delta_{f}H_{m}^{{}^{\circ}}\left(g\right)$ for the 2-acetyl-3-naphthol isomer.

(11) $$\Delta_{f}H_{m}^{{}^{\circ}}\left(g\right)=\;\Delta_{f}H_{m}^{{}^{\circ}}\left(\mathrm{cr}\right)+\;\Delta_{\mathrm{cr}}^{g}H_{m}^{{}^{\circ}}$$

During the combustion process of a given compound, all the energy stored in the bonds that bind atoms to each other is released because stable entities as carbon dioxide and water are formed (Equation (1)). It would be expected that isomers, like *o*-acetylnaphthol have similar combustion energy values (Table 1), and consequently similar crystal-phase enthalpy of formation values, however this does not happen, as can be seen by comparing the $\Delta_{f}H_{m}^{{}^{\circ}}\left(\mathrm{cr}\right)$ values for 1-acetyl-2-naphthol and 2-acetyl-1-napthol shown in Table 4. This difference reflects subtle structural variations caused by the exchange of the two substituents in positions 1 and 2. As the first approach, it can already be inferred that 1-acetyl-2-naphthol tends to be less stable, once it has the largest (most negative) heat of combustion. From the comparison of the $\Delta_{f}G_{m}^{{}^{\circ}}\left(\mathrm{cr}\right)$ is possible to conclude that the 2-acetyl-1-naphthol isomer is thermodynamically more stable in the crystalline phase.

At this stage, can the following question be asked: What will be the structural effect responsible for this difference? We will then analyze the enthalpy of sublimation, to see if there is any intermolecular interaction that is responsible for this difference. The values of the $\Delta_{\mathrm{cr}}^{g}H_{m}^{{}^{\circ}}$ at *T* = 298.15 K of 1-acetyl-2-naphthol and 2-acetyl-1-naphthol isomers are very close, with a difference of 2.1 kJ⋅mol^−1^ (Table 4). On the other hand, the entropic factor promotes additional stability to the crystal phase of 1-acetyl-2-naphthol leading to a high stability of this one, as can be confirmed by $\Delta_{\mathrm{cr}}^{g}G_{m}^{{}^{\circ}}$, thus making it more volatile.

Single crystal X-ray diffraction studies of 1-acetyl-2-naphthol at room temperature were performed by Hansen et al. [3]. The crystal system of 1-acetyl-2-naphthol (deposited with the Cambridge Crystallographic Data Centre with the number CCDC 151496) is a monoclinic with a non-standard space group P2_1_/n. According with these crystallographic data the acyl group is out of the naphthalene ring plane (about 24.5°) and the carbonyl group is slightly twisted towards the hydroxyl group, which structurally favors the formation of an IMHB, C=O⋯H-O-C. The same happens in the gas phase. Through the cut of the crystalline structure shown, it is possible to verify sets formed by 2 layers, in which each set is perpendicular to each other. Every set is formed by two parallel layers, in which the position of the molecules is inverted from one layer to the other.

The crystal system of 2-acetyl-1-naphthol (Crystallographic data deposited with the Cambridge Crystallographic Data Centre with the number CCDC 227364 [2]) is also a monoclinic but with a standard space group P2_1_/c. In this case, the molecule is planar, with the formation of an IMHB. 

Comparing the crystalline structures given [2,3] for the two isomers it appears that the 2-acetyl-1-naphthol has a more organized packaging, in which the molecules are arranged in parallel layers. There is no formation of intermolecular hydrogen bonds in the crystal phase for both *o*-acetylnaphthol isomers.

The results lead us to conclude that there is no significant difference in intermolecular interactions between the two compounds in the crystalline phase that justifies the observed difference in the $\Delta_{f}H_{m}^{{}^{\circ}}$ of both isomers. The next phase is the analysis of intramolecular interactions, using structural information from computational calculations obtained in the gas-phase. The *o*-acetylnaphthol isomers structurally differ in the position of the substituents; however, they have an IMHB in common, due to the close proximity and orientation of the oxygen of the acetyl group towards the hydrogen of the hydroxyl group.

The energy order of the IMHB present in the *o*-acetylnaphthol derivatives is 1-acetyl-2-naphtol < 2-acetyl-3-naphthol < 2-acetyl-1-naphthol. Likewise, this energy order is in line with the electronic density order (Table 3). Contrary to the normal trend [40], the IMHB of the 1-acetyl-2-naphthol isomer is the shortest and it has the lowest energy. The new ring structure formed as a result of the IMHB is not completely planar, once a twist of the carbonyl group with an out of plane bending of the C–C=O bond is observed, leading to a non-ideal overlap of the orbitals and a consequent weakening of the intramolecular hydrogen bond.

**Scheme 1:**
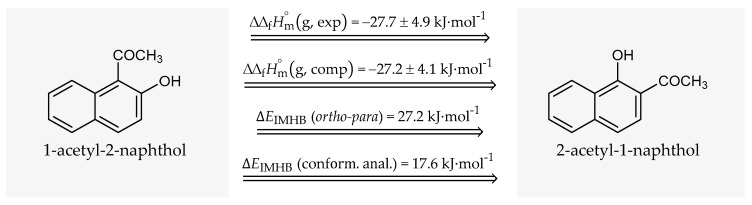
Relations on the energetics parameters obtained throughout the study for the title compounds.

Scheme 1 shows the differences between the energy parameters determined in the gas phase for the isomers 1-acetyl-naphthol and 2-acetyl-naphthol. The absolute values obtained for the $\Delta E_{\mathrm{IMHB}}\left(ortho-para\right)$, the $\Delta \Delta_{f}H_{m}^{{}^{\circ}}\left(g,\;\exp\right)\;$, and for the $\Delta \Delta_{f}H_{m}^{{}^{\circ}}\left(g,\;\mathrm{comp}\right)$ are in agreement. However, as already mentioned, in the *ortho-para* method used to calculate the $E_{\mathrm{IMHB}}$, the distortion effect of the acetyl group in the 1-acetyl-2-naphthol molecule is not accounted for. Thus, it can be considered that the value of 27.7 kJ⋅mol^−1^ obtained for these two isomers is equal to the sum of the effect of the $\Delta E_{\mathrm{IMHB}}\;(\mathrm{conform}.\;\mathrm{anal}.)$, 17.6 kJ⋅mol^−1^, and of the structural destabilization effect caused by the acetyl group, of 10.1 kJ⋅mol^−1^.

## 4. Materials and Methods 

### 4.1. Materials

Benzoic acid, CAS Registry No. 65-85-0, Standard Reference Material (SRM) 39j supplied by National Institute of Standards & Technology [41] was used in the calibration of the combustion calorimetry.

The samples of 1-acetyl-2-naphthol, CAS Registry No. 574-19-6, and 2-acetyl-1-naphthol, CAS Registry No. 711-79-5, were purchased from Aldrich Chemical Co., Darmstadt, Germany, with molar fraction purities 0.99.

The purification process of both compounds comprised the sublimation under reduced pressure. The purities of the samples were controlled by gas-liquid chromatography, using an Agilent 4890 apparatus equipped with an HP-5 column, cross-linked, 5% diphenyl and 95% dimethylpolysiloxane (15 m × 0.530 mm i. d. × 1.5 μm film thickness) and also by the ratio of carbon dioxide recovered in the combustion experiments. The samples of both compounds used in the study had molar fraction purities greater than 0.999.

### 4.2. Combustion Calorimetry

The combustion experiments of 1-acetyl-2-naphtol and 2-acetyl-1-naphthol were performed with an isoperibol calorimeter [19]. This calorimeter is equipped with a twin valve bomb, having an internal volume of 0.290 dm^3^ made of stainless steel (with a wall thickness of 1 cm).

The combustion experiments of the two crystal compounds were carried out in tablet form. In the case of the 2-acetyl-1-naphthol isomer, the tablets disintegrated with extreme ease, so it was necessary to use polyester bag of Melinex® (0.025 thickness) as combustion aid to facilitate their handling during weighing and also to ensure a complete combustion. Due to the incomplete combustion of the 1-acetyl-2-naphthol in some experiments, it was necessary to use *n*-hexadecane as combustion auxiliary. In each experiment, the sample together with the combustion aid were introduced in a platinum crucible inside the bomb and in contact with a cotton thread fuse attached to the platinum ignition wire (Goodfellows, *ϕ* = 0.05 mm). The combustion bomb was flushed and filled with pure oxygen ($x_{O_{2}}\geq 0.99995$) until reaching a pressure of 3.04 MPa and, approximately, 2900.0 g of water were introduced inside the calorimeter. The calorimeter temperature was measured to ± (1 × 10^−4^) K, at intervals of 10 seconds using a S10 four wire calibrated ultra-stable thermistor probe (Thermometrics, standard serial no. 1030) and recorded by a high sensitivity nanovolt/microohm meter (Agilent 34420 A) interfaced to a computer programmed to compute the adiabatic temperature change, Δ*T*_ad_. The samples were ignited at *T* = (298.150 ± 0.001) K, by the discharge of a 1400 μF capacitor through the platinum ignition wire.

The temperature profiles for each experiment were divided into three periods—fore-, main-, and after-periods—each one with 100 points at least. The energy of reaction refers to the initial temperature of 298.15 K. The accurate numerical calculation of the corrected temperature rise in the isoperibol calorimetry was carried out by means of the LABTERMO program [42] based in the method of calculation described by Coops et al. [43]. The electrical energy for the ignition Δ*U*(ign) was determined from the change in potential difference across a 1400 μF capacitor through the platinum ignition wire.

After the calorimetric measurements the combustion products were analyzed: the carbon dioxide formed was collected in absorption tubes [44] and calculated from the increase in weight of the tubes by multiplying it by the factor 1.0045, previously derived by Rossini [45]; the nitric acid formed, due to the presence of nitrogen residues in the oxygen used in the filling of the bomb, was determined by titration and the respective correction was based on −59.7 kJ·mol^−1^ (the molar energy of formation for 0.1 mol·dm^−3^ HNO_3_ (aq) from N_2_ (g), O_2_ (g) and H_2_O (l) [46]). 

The cotton thread used has an empirical formula CH_1.686_O_0.843_ and a specific energy of combustion 16,240 J·g^−1^ [43], a value previously confirmed in our laboratory. The values of standard combustion energy of the combustion aids used were: $\Delta_{c}u^{{}^{\circ}}$ = −(22,902 ± 5) J⋅g^−1^ for the Melinex®, reported by Skinner and Snelson [47] and meanwhile it has been confirmed in our laboratory;$\;\Delta_{c}u^{{}^{\circ}}$ = (−47,136.7 ± 2.3) J·g^−1^ for the *n*-hexadecane, measured in our laboratory, where the standard uncertainty quoted corresponds to the estimated standard deviation of the mean for ten experiments.

For the experiments with a small carbon soot residue formed during the combustion, the necessary energetic correction for its formation was based on standard molar energy of combustion of −33 kJ⋅mol^−1^ [46].

The value for the pressure coefficient of specific energy, ${\left(\partial u/\partial p\right)}_{T}$, at *T* = 298.15 K for both compounds was assumed to be −0.2 J·g^−1^·MPa^−1^, a common value for most organic solid compounds [48]. The mass of carbon dioxide, ${m(\mathrm{CO}}_{2},\;\mathrm{total})$, was calculated from the increase in mass of the absorption tubes by multiplying by the factor 1.0045, previously derived by Rossini [45]. The weights of the compounds and auxiliaries were made in a Mettler Toledo AT 201 balance, sensitivity ±(1×10^−5^) g, and the correction of weight in air to mass in vacuum was made using the corresponding specific density: 1.2 g⋅cm^−3^ for air, 1.977 g⋅cm^−3^ for carbon dioxide, 1.20 g⋅cm^−3^ for benzoic acid, 1.5 g⋅cm^−3^ for cotton thread fuse, 1.361 g⋅cm^−3^ for 1-acetyl-2-naphthol [3] and 1.365 g⋅cm^−3^ for 2-acetyl-1-naphthol [2]. The relative atomic masses used throughout this study are those recommended by IUPAC [49], and the value of the molar gas constant was *R* = 8.314462618 J·mol^−1^·K^−1^ [24].

Calculations for the standard massic energy of combustion, $\Delta_{c}u^{{}^{\circ}}$, and for the corrections to the standard state, $\Delta U_{\Sigma }$, follow the procedure given by Hubbard et al. [20].

The calorimetric system was calibrated using benzoic acid Standard Reference Material (SRM) 39j supplied by National Institute of Standards & Technology, having a massic energy of combustion of (−26,434 ± 3) J·g^−1^, when burned under the standard bomb conditions [41]. Calibration experiments were carried out under the same conditions as the compound experiments. The calibration results were corrected to give the energy equivalent, ε(cal), corresponding to the average mass of water added to the calorimeter of 2900.0 g. The values of the ε(cal) used in the combustion calorimetry experiments were: ε(cal) = (15,553.0 ± 1.1) J⋅g^−1^ in the study of 1-acetyl-2-naphthol and ε(cal) = (15,551.2 ± 1.7) J⋅g^−1^ in the study of 2-acetyl-1-naphthol; the standard uncertainty quoted is the estimated standard deviation of the mean for six experiments.

### 4.3. Knudsen Effusion Method

A full description of the main features of the apparatus together with the results obtained with five test substances (benzoic acid, phenanthrene, anthracene, benzanthrone, and 1,3,5-triphenylbenzene) have been reported [23].

The Knudsen effusion apparatus used enables the simultaneous operation of nine aluminum cells divided into three sets, each with different effusion orifice areas. The effusion orifices, made in platinum foil of (0.0125 ± 0.001) mm thickness, have the following areas: A_0_(S1) = A_0_(S2) = A_0_(S3) = (0.636 ± 0.004) mm^2^, A_0_(M4) = A_0_(M5) = A_0_(M6) = (0.785 ± 0.004) mm^2^, A_0_(L7) = A_0_(L8) = A_0_(L9) = (0.985 ± 0.004) mm^2^, where the uncertainties were calculated by the RSS method. The Clausing factors of the effusion orifices were calculated as $\omega_{0}$ = {1 + (*l*/2*r*)}^−1^, where *l* is the thickness of the platinum foil and *r* is the radius of the orifices, yielding the results 0.986, 0.988, and 0.989 for the orifices of the series S, M, and L, respectively. The system has three aluminum blocks that can be kept at different temperatures from each other. Each block has three cylindrical holes where are placed one cell of each set.

All the weighing were made in a Balance Mettler Toledo EA 163 balance, sensitivity ±(1 × 10^−5^) g.

### 4.4. Computational Studies

The energies of the molecules were calculated using the Gaussian-3 theory at the G3(MP2)//B3LYP [18] level, as included in the Gaussian 03 suite of programs [50]. The geometries are obtained at the B3LYP/6-31G(*d*) level, as well as the zero-point energies (scaled by 0.96). The single point energies calculations are made at the QCISD(T,FC)/6-31G(*d*) and MP2(FC)/G3MP2large levels.

## 5. Conclusions

The knowledge of the structural-energetic relations was achieved combining experimental and computational studies. 2-Acetyl-1-naphthol is thermodynamically more stable than 1-acetyl-2-naphthol isomer, both in the crystalline and gas phases. The energetic difference between the two isomers, of 27.7 kJ⋅mol^−1^, is due to the energy of the intramolecular hydrogen bond, which in the case of 1-acetyl-2-naphthol is weaker, and also due to the structural destabilizing effect present in the 1-acetyl-2-naphthol caused by the distortion of the acetyl group.

The excellent agreement between the experimental and computational values of $\Delta_{f}H_{m}^{{}^{\circ}}\left(g\right)$ obtained for the title compounds, validates the estimation method used, and consequently gives confidence to the $\Delta_{f}H_{m}^{{}^{\circ}}\left(g\right)$ derived for the isomer 2-acetyl-3-naphthol, −213.1 ± 3.5 kJmol^−1^.

The isomers can be ordered in ascending order of stabilization in the gaseous phase this way: 1-acetyl-2-napthol, 2-acetyl-3-napthol, and 2-acetyl-1-naphthol.

From the gas phase tautomeric keto ↔ enol equilibria study of the *o*-acetylnaphthol isomers, it was concluded that the enol form is the most stable in the gas phase.

## Figures and Tables

**Table 1:** Derived standard (*p* = 0.1 MPa) molar values from combustion calorimetry experiments for 1-acetyl-2-naphthol and 2-acetyl-1-naphthol in the crystalline phase, at *T* = 298.15 K.

Compound	$\Delta_{c}U_{m}^{{}^{\circ}}(\mathrm{cr})/\mathrm{kJ}\cdot {\mathrm{mol}}^{-1}$	$\Delta_{c}H_{m}^{{}^{\circ}}(\mathrm{cr})/\mathrm{kJ}\cdot {\mathrm{mol}}^{-1}$	$\Delta_{f}H_{m}^{{}^{\circ}}(\mathrm{cr})/\mathrm{kJ}\cdot {\mathrm{mol}}^{-1}$
1-acetyl-2-naphthol	−5850.2 ± 1.6 ^1^	−5853.9 ± 1.6 ^1^	−297.3 ± 2.2 ^2^
2-acetyl-1-naphthol	−5824.7 ± 3.0 ^1^	−5828.4 ± 3.0 ^1^	−322.9 ± 3.4 ^2^

^1^ The uncertainty corresponds to the expanded uncertainty determined from the combined standard uncertainty (which include the contribution of the calibration with benzoic acid and the combustion aids used) and the coverage factor *k* = 2 (0.95 level of confidence) [22]; ^2^ The uncertainty corresponds to the expanded uncertainty determined from the combined standard uncertainty (which include the contribution of the species involved in the combustion reaction, Equation (1)) and the coverage factor *k* = 2 (0.95 level of confidence) [22].

**Table 2:** Standard ($p^{{}^{\circ}}$ = 0.1 MPa) molar enthalpies, $\Delta_{\mathrm{cr}}^{g}H_{m}^{{}^{\circ}}$, entropies, $\Delta_{\mathrm{cr}}^{g}S_{m}^{{}^{\circ}}$, and Gibbs energies, $\Delta_{\mathrm{cr}}^{g}G_{m}^{{}^{\circ}}$, of sublimation, and vapor pressure, *p*, at *T* = 298.15 K, for 1-acetyl-2-naphthol and 2-acetyl-1-naphthol ^1^

Compound	$\Delta_{\mathrm{cr}}^{g}H_{m}^{{}^{\circ}}/\mathrm{kJ}\cdot {\mathrm{mol}}^{-1}$	$\Delta_{\mathrm{cr}}^{g}S_{m}^{{}^{\circ}}/J\cdot K^{-1}\cdot {\mathrm{mol}}^{-1}$	$\Delta_{\mathrm{cr}}^{g}G_{m}^{{}^{\circ}}/\mathrm{kJ}\cdot {\mathrm{mol}}^{-1}$	p/Pa
1-acetyl-2-naphthol	100.9 ± 0.6	212.3 ± 1.9	37.6 ± 0.8	2.58 × 10^−2^
2-acetyl-1-naphthol	98.8 ± 0.5	200.0 ± 1.5	39.2 ± 0.7	1.37 × 10^−2^

^1^ The uncertainties quoted are the combined standard uncertainties (0.95 level of confidence).

**Table 3:** Energy and respective length of the intramolecular hydrogen bond (IMHB) present in the *o*-acetylnaphthol isomers in the gas phase obtained by computational calculation

Compound	IMHB Length/10^−10^ m	*ρ*_BCP_/a.u.	*E*_IMHB_/kJ⋅mol^−1^	
			*ortho-para* Method	Conformational Analysis
1-acetyl-2-naphthol	1.59	6.56 × 10^−3^	16.4	25.9
2-acetyl-1-naphthol	1.64	3.63 × 10^−2^	43.6	43.5
2-acetyl-3-naphthol	1.70	3.27 × 10^−2^	31.5	31.3

**Table 4:** Compilation of the thermodynamic results obtained for *o*-acetylnaphthol isomers.

	1-acetyl-2-naphthol	2-acetyl-1-naphthol	2-acetyl-3-naphthol
**Gaseous Phase**			
$\Delta_{f}H_{m}^{{}^{\circ}}\left(g\right)$ **/kJ** **⋅mol ^−1^**	−198.0 ± 3.5 ^1^ −196.4 ± 2.3 ^2^	−225.2 ± 3.5 ^1^ −224.1 ± 3.4 ^2^	−213.1 ± 3.5 ^1^
$\Delta_{f}S_{m}^{{}^{\circ}}\left(g\right)$ **/J** **⋅K ^−1^** **⋅mol ^−1^**	−503.1 ^1,3^	−499.2 ^1,3^	−496.7 ^1,3^
$\Delta_{f}G_{m}^{{}^{\circ}}\left(g\right)$ **/kJ** **⋅mol ^−1^**	−48.0 ^1,3^	−76.4 ^1,3^	−65.0 ^1,3^
**Condensed Phase**			
$\Delta_{f}H_{m}^{{}^{\circ}}\left(\mathrm{cr}\right)$ **/kJ** **⋅mol ^−1^**	−297.3 ± 2.2 ^2^	−322.9 ± 3.4 ^2^	—
$\Delta_{f}S_{m}^{{}^{\circ}}\left(\mathrm{cr}\right)$ **/J** **⋅K ^−1^** **⋅mol ^−1^**	−715.4 ^4^	−699.2 ^4^	—
$\Delta_{f}G_{m}^{{}^{\circ}}\left(\mathrm{cr}\right)$ **/kJ** **⋅mol ^−1^**	−84.0 ^5^	−114.4 ^5^	—
**Phase transition crystal to gas**			
$\Delta_{\mathrm{cr}}^{g}H_{m}^{{}^{\circ}}$ **/kJ** **⋅mol ^−1^**	100.9 ± 0.6 ^2^	98.8 ± 0.5 ^2^	—
$\Delta_{\mathrm{cr}}^{g}S_{m}^{{}^{\circ}}$ **/J** **⋅K ^−1^** **⋅mol ^−1^**	212.3 ± 1.9 ^2^	200.0 ± 1.5 ^2^	—
$\Delta_{\mathrm{cr}}^{g}G_{m}^{{}^{\circ}}$ **/kJ** **⋅mol ^−1^**	37.6 ± 0.8 ^2^	39.2 ± 0.7 ^2^	—

^1^ Derived from computational studies (Table S9 in Supplementary Materials); ^2^ Derived from experimental studies, using Equation (11); ^3^ The method of calculation is given in Supplementary Materials in Tables S5–S7; ^4^ Calculated from $\Delta_{f}S_{m}^{{}^{\circ}}\left(\mathrm{cr}\right)\;=\;\Delta_{f}S_{m}^{{}^{\circ}}\left(g\right)-\Delta_{\mathrm{cr}}^{g}S_{m}^{{}^{\circ}}$; ^5^ Calculated from $\Delta_{f}G_{m}^{{}^{\circ}}\left(\mathrm{cr}\right)\;=\;\Delta_{f}H_{m}^{{}^{\circ}}\left(\mathrm{cr}\right)-\;T\Delta_{f}S_{m}^{{}^{\circ}}\left(\mathrm{cr}\right)$.

## Supplementary Materials

Table S1. Combustion results and standard (*p*^°^ = 0.1 MPa) energies of combustion, at *T* = 298.15 K, for 1-acetyl-2-naphthol (in the crystalline state); Table S2. Combustion results and standard (*p*^°^ = 0.1 MPa) energies of combustion, at *T* = 298.15 K, for 2-acetyl-1-naphthol (in the crystalline state); Table S3. Knudsen effusion experimental results for 1-acetyl-2-naphthol and 2-acetyl-1-naphthol; Table S4. Parameters of the Clausius-Clapeyron equation for the two *o*-acetylnaphthol isomers studied and calculated values for the enthalpy and entropy at the mean temperature, 〈*T*〉, of the experiment; Tables S5–S7. Absolute standard enthalpies, $H_{298.15\;K}^{{}^{\circ}}$, and entropies, $S_{298.15\;K}^{{}^{\circ}}$, obtained by G3(MP2)//B3LYP composite method and the corresponding derived gas-phase standard molar enthalpies, $\Delta_{f}H_{m}^{{}^{\circ}}\left(g\right)$, entropies, $\Delta_{f}S_{m}^{{}^{\circ}}\left(g\right)$, and Gibbs energy of formation, $\Delta_{f}G_{m}^{{}^{\circ}}\left(g\right)$, at *T* = 298.15 K, as well as the conformational composition, χ_I_, obtained for for, respectively, 1-acetyl-2-naphthol, 2-acetyl-1-naphthol, and 2-acetyl-3-naphthol; Table S8. G3(MP2)//B3LYP enthalpies, $H_{298.15\;K}^{{}^{\circ}}$, and experimental gas-phase standard (*p*^°^ = 0.1 MPa) molar enthalpies of formation, $\Delta_{f}H_{m}^{{}^{\circ}}\left(g\right)$, at *T* = 298.15 K, for *o*-acetylnaphthol isomers and for the auxiliary species; Table S9. Group substitution reactions for 1-acetyl-2-naphthol, 2-acetyl-1-naphthol, and 2-acetyl-3-naphthol and corresponding calculated values for the enthalpies of formation, $\Delta_{f}H_{m}^{{}^{\circ}}\left(g\right)$, in the gaseous-phase, at *T* = 298.15 K; Table S10. Gibbs energies, $G_{298.15\;K}^{{}^{\circ}}$, obtained by G3(MP2)//B3LYP composite method for *o*-acetylnaphthol (enol-tautomers) and the matching keto-tautomers, and the theoretically predicted gas-phase standard molar Gibbs energies, $\Delta_{r}G_{m}^{{}^{\circ}}\left(g\right)$, for the enol ↔ keto equilibrium, at *T* = 298.15 K, with the corresponding fractions (*x*) of the two tautomers.
