# Supplementary material for: Structural and Energetic Insights on Two Dye Compounds: 1-Acetyl-2-Naphthol and 2-Acetyl-1-Naphthol
Source: Molecules. 2020 Aug 22;25(17):3827. doi: 10.3390/molecules25173827 (PMC7504395; doi:10.3390/molecules25173827)
Supplement: Supplementary file 1 [file molecules-25-03827-s001.pdf]

Structural and energetic insights on two dye compounds: 1-acetyl-2-naphthol and 2-acetyl-1-naphthol

Vera L. S. Freitas [1,*], Maria D. M. C. Ribeiro da Silva [1]

[1]Centro de Investigação em Química da Universidade do Porto (CIQUP), Department of Chemistry and Biochemistry, Faculty of Science, University of Porto, Rua do Campo Alegre, P-4169-007 Porto, Portugal;

mdsilva@fc.up.pt (M.D.M.C.R.S.)
*Correspondence: vera.freitas@fc.up.pt; Tel.: +35-122-040-2538

Received: date; Accepted: date; Published: date

Table of Contents

page

Table S1. Combustion results and standard ($p°$ = 0.1 MPa) energies of combustion, at T = 298.15 K, for 1-acetyl-2-naphthol (in the crystalline state) A3

Table S2. Combustion results and standard ($p°$ = 0.1 MPa) energies of combustion, at T = 298.15 K, for 2-acetyl-1-naphthol (in the crystalline state) A4

Table S3. Knudsen effusion experimental results for 1-acetyl-2-naphthol and 2-acetyl-1-naphthol A5

Table S4. Parameters of the Clausius-Clapeyron equation for the two *o*-acetylnaphthol isomers studied and calculated values for the enthalpy and entropy at the mean temperature, $\langle T \rangle$, of the experiment A6

Table S5. Absolute standard enthalpies, $H^{\circ}_{298.15\ \mathrm{K}}$, and entropies, $S^{\circ}_{298.15\ \mathrm{K}}$, obtained by G3(MP2)//B3LYP composite method for 1-acetyl-2-naphthol and the corresponding derived gas-phase standard molar enthalpies, $\Delta_f H^{\circ}_m$(g), entropies, $\Delta_f S^{\circ}_m$(g), and Gibbs energy of formation, $\Delta_f G^{\circ}_m$(g), at T = 298.15 K, and the conformational composition, χ_i. A7

Table S6. Absolute standard enthalpies, $H^{\circ}_{298.15\ \mathrm{K}}$, and entropies, $S^{\circ}_{298.15\ \mathrm{K}}$, obtained by G3(MP2)//B3LYP composite method for 2-acetyl-1-naphthol and the corresponding derived gas-phase standard molar enthalpies, $\Delta_f H^{\circ}_m$(g), entropies, $\Delta_f S^{\circ}_m$(g), and Gibbs energy of formation, $\Delta_f G^{\circ}_m$(g), at T = 298.15 K, and the conformational composition, χ_i. A8

Table S7. Absolute standard enthalpies, $H^{\circ}_{298.15\ \mathrm{K}}$, and entropies, $S^{\circ}_{298.15\ \mathrm{K}}$, obtained by G3(MP2)//B3LYP composite method for 2-acetyl-3-naphthol and the corresponding derived gas-phase standard molar enthalpies, $\Delta_f H^{\circ}_m$(g), entropies, $\Delta_f S^{\circ}_m$(g), and Gibbs energy of formation, $\Delta_f G^{\circ}_m$(g), at T = 298.15 K, and the conformational composition, χ_i. A9

Table S8. G3(MP2)//B3LYP enthalpies, $H^{\circ}_{298.15\mathrm{K}}$, and experimental gas-phase standard ($p°$ = 0.1 MPa) molar enthalpies of formation, $\Delta_f H^{\circ}_m$(g), at T = 298.15 K, for *o*-acetylnaphthol isomers and for the auxiliary species A10

Table S9 Group substitution reactions for 1-acetyl-2-naphthol, 2-acetyl-1-naphthol, and 2-acetyl-3-naphthol and corresponding calculated values for the enthalpies of formation, $\Delta_f H^\circ_m$(g), in the gaseous-phase, at T = 298.15 K A12

Table S10. Gibbs energies, $G^\circ_{298.15\ K}$, obtained by G3(MP2)//B3LYP composite method for *o*-acetylnaphthol (enol-tautomers) and the matching keto-tautomers, and the theoretically predicted gas-phase standard molar Gibbs energies, $\Delta_r G^\circ_m(g)$, for the enol↔keto equilibrium, at T = 298.15 K, with the corresponding fractions (x) of the two tautomers A13

References A14

S1. Combustion calorimetry – Energy of combustion

Table S1. Combustion results and standard ($p°$ = 0.1 MPa) energies of combustion, at T = 298.15 K, for 1-acetyl-2-naphthol (in the crystalline state).[1]

**Experiment**	**1**	**2**	**3**	**4**	**5**	**6**
m (CO_2, total) / g	2.20222	2.37483	2.48844	2.22080	2.21169	2.34895
m (cpd) / g	0.63551	0.63821	0.68512	0.57341	0.59150	0.64304
m (fuse) / g	0.00301	0.00272	43.69	0.00327	50.34	0.00325
m (n-hexadecane) / g	0.12747	0.17956	0.17402	0.18963	0.17014	0.16740
m (carbon) / g	0.00040	0.00013	0.00005	0.00012	0	0.00017
T_i / K	298.15066	298.14990	298.15039	298.15107	298.15072	298.15083
T_f / K	299.87963	300.03672	300.11320	299.94170	299.91761	300.01105
ΔT_{ad} / K	1.67221	1.83549	1.91417	1.73630	1.71224	1.80774
ε_f / ($J\cdot K^{-1}$)	16.18	16.48	16.57	16.37	16.31	16.42
Δm (H_2O) / g	–1.0	–0.6	–1.6	–1.9	1.7	2.0
$-\Delta U$ (IBP) / J	26027.33	28572.34	29789.42	27018.69	26669.94	28160.03
ΔU (carbon) / J	13.20	4.29	1.65	3.96	0	5.61
$-\Delta U$ (n-hexadecane) / J	6008.60	8463.96	8202.95	8938.33	8019.81	7890.48
$-\Delta U$ (fuse) / J	48.88	44.17	43.69	53.10	50.34	52.78
ΔU (HNO_3) / J	1.43	0.67	1.18	2.60	1.00	1.82
ΔU (ign) / J	0.61	0.68	0.57	0.60	0.63	0.56
ΔU_{Σ} / J	15.42	16.41	17.50	14.96	15.08	16.32
$-\Delta_c u^o$ / ($J\cdot g^{-1}$)	31417.60	31418.22	31418.95	31414.97	31417.94	31419.88
% CO_2	100.072	100.002	99.987	100.029	100.031	100.000
$\langle -\Delta_c u^o \rangle$ = (31417.9 ± 0.7) $J\cdot g^{-1}$ [2]						
$\langle -\Delta_c U^o \rangle$ = (5850.2 ± 1.6) $J\cdot mol^{-1}$ [3]						
$\langle$% $CO_2\rangle$= (100.02 ± 0.01) [2]						

[1] The symbols presented in this table have the following meaning: m(CO_2, total), total mass of carbon dioxide recovered; m(cpd), mass of compound considering the carbon dioxide mass fraction recovery; m(fuse), mass of fuse (cotton); m(n-hexadecane), mass of n-hexadecane used as auxiliary combustion material; m(carbon), mass of carbon residue formed; T_i, initial temperature rise; T_f, final temperature rise; ΔT_{ad}, corrected temperature rise; ε_f, energy equivalent of the contents in the final state; Δm(H_2O), deviation of mass of water added to the calorimeter from 2900.0 g; ΔU(IBP), internal energy associated to the isothermal combustion reaction under actual bomb conditions (eq. 2 of manuscript); ΔU(carbon), energy of combustion of carbon residue; ΔU(n-hexadecane), energy of combustion of n-hexadecane; ΔU(fuse), energy of combustion of the fuse (cotton); ΔU(HNO_3), energy correction for the nitric acid formation; ΔU(ign), electric energy for the ignition; ΔU_{Σ}, standard state correction; $\Delta_c u^o$, standard ($p°$ = 0.1 MPa) massic energy of combustion for the burnt compound; % CO_2, percentage of carbon dioxide recovered;

[2] The standard uncertainty corresponds to the estimated standard deviation of the mean for six experiments;

[3] The uncertainty corresponds to the expanded uncertainty determined from the combined standard uncertainty (which include the contribution of the calibration with benzoic acid and the combustion aids used) and the coverage factor k = 2 (0.95 level of confidence).

Table S2. Combustion results and standard ($p°$ = 0.1 MPa) energies of combustion, at T = 298.15 K, for 2-acetyl-1-naphthol (in the crystalline state).[1]

Experiment	1	2	3	4	5	6
m (CO_2, total) / g	1.82499	1.43031	1.31780	1.73618	0.83469	1.37977
m (cpd) / g	0.60087	0.42347	0.41928	0.56614	0.24891	0.44967
m (fuse) / g	0.00327	0.00289	0.00263	0.00318	0.00163	0.00245
m (carbon) / g	0.00007	0.00010	0.00019	0.00011	0.00017	0.00010
m (Melinex) / g	0.05056	0.09823	0.05462	0.05492	0.05533	0.04403
T_i / K	298.14956	298.15072	298.15152	298.15121	298.15116	298.15060
T_f / K	299.50067	299.23013	299.18852	299.43702	298.82576	299.18811
ΔT_{ad} / K	1.28693	0.99902	0.92555	1.22258	0.58327	0.97043
ε_f / (J·K^{-1})	15.54	15.16	15.09	15.47	14.67	15.10
Δm (H_2O) / g	-0.3	1.3	2.1	0.3	0.1	1.2
$-\Delta U$ (IBP) / J	20031.02	15555.33	14414.31	19031.83	9078.16	15109.67
$-\Delta U$ (Melinex) / J	1158.00	2249.75	1250.98	1257.83	1267.20	1008.37
ΔU (carbon) / J	2.31	3.30	6.27	3.63	5.61	3.30
$-\Delta U$ (fuse) / J	53.10	46.93	42.71	51.64	26.47	39.79
$-\Delta U$ (HNO_3) / J	2.11	0.41	2.15	0.69	0.09	0.53
ΔU (ign) / J	0.67	1.21	1.20	1.20	1.19	1.21
ΔU_Σ / J	13.64	10.57	9.55	12.93	5.88	9.96
$-\Delta_c u^o$ / (J·g^{-1})	31298.75	31291.40	31280.27	31286.20	31272.87	31254.74
% CO_2	100.008	99.996	99.984	99.902	99.984	100.028

$\langle -\Delta_c u^o \rangle$ = (31280.7 ± 6.3) J·g^{-1} [2]

$\langle -\Delta_c U^o \rangle$ = (5824.7 ± 3.0 ± 6.3) J·mol^{-1} [3]

$\langle \% \, CO_2 \rangle$= (99.98 ± 0.02) [2]

[1] The symbols presented in this table have the same meaning of the ones presented in the above table, adding the following symbols that have not yet appeared: m(Melinex), mass of Melinex used as auxiliary combustion material; ΔU(Melinex), energy of combustion of Melinex.

[2] The standard uncertainty corresponds to the estimated standard deviation of the mean for six experiments;

[3] The uncertainty corresponds to the expanded uncertainty determined from the combined standard uncertainty (which include the contribution of the calibration with benzoic acid and the combustion aids used) and the coverage factor k = 2 (0.95 level of confidence).

S2. Knudsen mass-loss effusion method – Enthalpy of sublimation

Table S3. Knudsen effusion experimental results for 1-acetyl-2-naphthol and 2-acetyl-1-naphthol.[1]

T / K [2]	t / s	Orificies [3]	m / mg [4]			p / Pa [5]		
			m_S	m_M	m_L	p_S	p_M	p_L
**1-acetyl-2-naphthol**								
306.19	32974	S3-M6-L9	5.21	6.47	8.16	0.0738	0.0741	0.0744
308.33	32974	S2-M5-L8	6.74	8.40	10.44	0.0958	0.0966	0.0956
310.13	32974	S1-M4-L7	8.66	10.51	13.06	0.1235	0.1212	0.1199
312.19	23899	S3-M6-L9	8.00	9.94	12.34	0.1579	0.1587	0.1568
314.39	23899	S2-M5-L8	10.16	12.73	15.58	0.2012	0.2039	0.1987
316.13	23899	S1-M4-L7	12.83	15.72	19.54	0.2548	0.2525	0.2499
318.18	12958	S3-M6-L9	8.94	10.90	13.70	0.3285	0.3240	0.3242
320.45	12958	S2-M5-L8	11.21	13.94	17.22	0.4134	0.4158	0.4090
322.13	12958	S1-M4-L7	14.20	17.35	21.33	0.5250	0.5189	0.5079
324.20	10038	S3-M6-L9	13.81	16.99	21.24	0.6613	0.6581	0.6550
326.29	10038	S2-M5-L8	17.48	21.39	26.51	0.8397	0.8312	0.8201
328.11	10038	S1-M4-L7	21.73	26.71	32.20	1.0468	1.0408	0.9989
**2-acetyl-1-naphthol**								
312.19	39950	S3-M6-L9	6.79	8.33	10.47	0.0802	0.0796	0.0796
314.41	39950	S2-M5-L8	8.70	10.60	13.16	0.1031	0.1016	0.1004
316.13	39950	S1-M4-L7	10.74	13.18	16.19	0.1276	0.1267	0.1239
318.17	23744	S3-M6-L9	8.00	9.86	12.48	0.1604	0.1599	0.1612
320.38	23744	S2-M5-L8	10.10	12.57	15.55	0.2032	0.2046	0.2015
322.14	23744	S1-M4-L7	12.63	15.45	18.92	0.2549	0.2522	0.2459
324.18	14137	S3-M6-L9	9.32	11.55	14.43	0.3169	0.3176	0.3159
326.33	14137	S2-M5-L8	11.61	14.50	17.88	0.3960	0.4001	0.3928
328.13	14137	S1-M4-L7	14.49	17.67	21.84	0.4956	0.4889	0.4811
330.19	7536	S3-M6-L9	9.70	11.84	14.82	0.6244	0.6165	0.6143
332.19	10147	S3-M6-L9	16.02	19.77	24.70	0.7681	0.7668	0.7627
334.12	7536	S1-M4-L7	14.75	18.04	22.00	0.9551	0.9448	0.9174
336.12	10147	S1-M4-L7	24.21	29.67	36.35	1.1677	1.1576	1.1291

[1]The subscripts S, M, L of the variables m (mass effused) and p (vapor pressure) correspond to the results obtained through the small (S1, S2, and S3), the medium (M4, M5, and M6) and the large (L7, L8, and L9) effusion orifices, respectively;

[2]The standard uncertainty of the measured temperature is ± ($1{\cdot}10^{-2}$) K;

[3]The areas, A_0, and transmission probability Clausing factors, ω_0, for each effusion orifice are: A_0(S1) = A_0(S2) = A_0(S3) = (0.636 ± 0.004) mm^2, A_0(M4) = A_0(M5) = A_0(M6) = (0.785 ± 0.004) mm^2, A_0(L7) = A_0(L8) = A_0(L9) = (0.985 ± 0.004) mm^2; ω_0 (S1) = ω_0 (S2) = ω_0 (S3) = 0.986, ω_0 (M4) = ω_0 (M5) = ω_0 (M6) = 0.988, ω_0 (L7) = ω_0 (L8) = ω_0 (L9) = 0.989.

[4]The standard uncertainty is ± ($1{\cdot}10^{-2}$) mg according to manufacturer's specifications (Analytical Balance Mettler Toledo EA 163);

[5]The standard uncertainty is ± ($3{\cdot}10^{-2}$) Pa.

Table S4. Parameters of the Clausius-Clapeyron equation for the two *o*-acetylnaphthol isomers studied and calculated values for the enthalpy and entropy at the mean temperature, $\langle T \rangle$, of the experiment.

Orifices	a	b	$p(\langle T \rangle)$ / Pa	$\Delta_{cr}^{g} H_m (\langle T \rangle)$ / kJ·mol^{-1}	$\Delta_{cr}^{g} S_m (\langle T \rangle, p\langle T \rangle)$/ J·K^{-1}·mol^{-1}
**1-acetyl-2-naphthol $\langle T \rangle$ = 317.15 K**					
S1-S2-S3	37.0 ± 0.5 [1]	10629 ± 174 [1]	0.298	100.8 ± 1.2 [5]	317.8 ± 3.8 [5]
M4-M5-M6	36.8 ± 0.3 [1]	10607 ± 214 [1]	0.286	100.3 ± 0.8 [5]	316.3 ± 2.5 [5]
L7-L8-L9	36.5 ± 0.4 [1]	10601 ± 149 [1]	0.278	99.6 ± 1.1 [5]	314.0 ± 3.5 [5]
Global results	36.8 ± 0.2 [2]	10612 ± 102 [2]	0.297	100.2 ± 0.6 [5]	315.9 ± 1.9 [5]
**2-acetyl-1-naphthol $\langle T \rangle$ = 324.16 K**					
S1-S2-S3	35.2 ± 0.3 [3]	11793 ± 108 [3]	0.307	98.1 ± 0.9 [5]	302.6 ± 2.8 [5]
M4-M5-M6	35.2 ± 0.2 [3]	11790 ± 64 [3]	0.310	98.0 ± 0.5 [5]	302.3 ± 1.5 [5]
L7-L8-L9	35.0 ± 0.4 [3]	11731 ± 118 [3]	0.304	97.5 ± 1.0 [5]	300.8 ± 3.1[5]
Global results	35.2 ± 0.2 [4]	11772 ± 63 [4]	0.328	97.9 ± 0.5 [5]	302.0 ± 1.5 [5]

[1]The standard uncertainty were obtained by multiplying the standard error of the fitting parameters by k=2.228, that corresponds to the *t*-distribution value for 0.95 level of confidence and 10 degrees of freedom.

[2]The standard uncertainty were obtained by multiplying the standard error of the fitting parameters by k=2.032, that corresponds to the *t*-distribution value for 0.95 level of confidence and 34 degrees of freedom.

[3]The standard uncertainty were obtained by multiplying the standard error of the fitting parameters by k=2.201, that corresponds to the *t*-distribution value for 0.95 level of confidence and 11 degrees of freedom.

[4]The standard uncertainty were obtained by multiplying the standard error of the fitting parameters by k=2.026, that corresponds to the *t*-distribution value for 0.95 level of confidence and 37 degrees of freedom.

[5]The uncertainties quoted are the combined standard uncertainties (0.95 level of confidence).

S3. Computational studies

Table S5. Absolute standard enthalpies, $H^\circ_{298.15\,\mathrm{K}}$, and entropies, $S^\circ_{298.15\,\mathrm{K}}$, obtained by G3(MP2)//B3LYP composite method for 1-acetyl-2-naphthol and the corresponding derived gas-phase standard molar enthalpies, $\Delta_\mathrm{f}H^\circ_\mathrm{m}(\mathrm{g})$, entropies, $\Delta_\mathrm{f}S^\circ_\mathrm{m}(\mathrm{g})$, and Gibbs energy of formation, $\Delta_\mathrm{f}G^\circ_\mathrm{m}(\mathrm{g})$, at T = 298.15 K, and the conformational composition, χ_i. 1 a. u. (Hartree) corresponds to 2625.50 kJ·mol^{-1}.

Conformation[1]	$H^\circ_{298.15\mathrm{K}}$ [2] / a.u.	$\Delta_\mathrm{f}H^\circ_\mathrm{m}(\mathrm{g})$ [3] / kJ·mol^{-1}	$S^\circ_{298.15\mathrm{K}}$ [4] / J·K^{-1}·mol^{-1}	$\Delta_\mathrm{f}S^\circ_\mathrm{m}(\mathrm{g})$ [5] / J·K^{-1}·mol^{-1}	$\Delta_\mathrm{f}G^\circ_\mathrm{m}(\mathrm{g})$ [6] / kJ·mol^{-1}	χ_i [7]
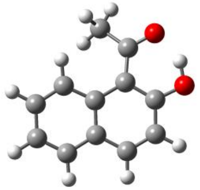	–612.814664	–198.0 ± 3.5	424.35	–503.1	–48.0	1.000
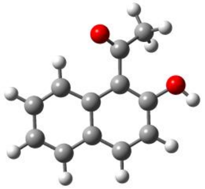	–612.804792	–172.1 ± 3.5	439.96	–487.5	–26.8	0
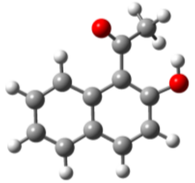	–612.802595	–166.3 ± 3.5	437.01	–490.4	–20.1	0

[1]Spheres color code: grey, C; red, O; white, H.
[2]Obtained from G3(MP2)//B3LYP method [1]
[3]Estimated from 16 working reactions presented on Table 7 of manuscript;
[4]Obtained from B3LYP/6-31G(*d*) method for a frequency factor scale of 1.0029 [2];
[5]Calculated from $\Delta_\mathrm{f}S^\circ_\mathrm{m}(\mathrm{g}) = S^\circ_{298.15\mathrm{K}}$ (conformer i) $- \sum S^\circ_{298.15\mathrm{K}}$ (elements), considering the standard absolute entropy elements values, at 298.15 K, $S^\circ_{298.15\mathrm{K}}(\mathrm{H_2, g})$ = 130.680 J·K^{-1}·mol^{-1}, $S^\circ_{298.15\mathrm{K}}(\mathrm{C, graphite})$ = 5.740 J·K^{-1}·mol^{-1} and $S^\circ_{298.15\mathrm{K}}(\mathrm{O_2, g})$ = 205.147 J·K^{-1}·mol^{-1} taken from ref. [3];
[6]Calculated from $\Delta_\mathrm{f}G^\circ_\mathrm{m}(\mathrm{g}) = \Delta_\mathrm{f}H^\circ_\mathrm{m}(\mathrm{g}) - T\Delta_\mathrm{f}S^\circ_\mathrm{m}(\mathrm{g})$;
[7]Calculated from $\chi_i = e^{-[\Delta_\mathrm{f}G^\circ_\mathrm{m}(\mathrm{g})/RT]}/\sum_i^n e^{-[\Delta_\mathrm{f}G^\circ_\mathrm{m}(\mathrm{g})/RT]}$.

Table S6. Absolute standard enthalpies, $H^{\circ}_{298.15\,\mathrm{K}}$, and entropies, $S^{\circ}_{298.15\,\mathrm{K}}$, obtained by G3(MP2)//B3LYP composite method for 2-acetyl-1-naphthol and the corresponding derived gas-phase standard molar enthalpies, $\Delta_f H^{\circ}_m(\mathrm{g})$, entropies, $\Delta_f S^{\circ}_m(\mathrm{g})$, and Gibbs energy of formation, $\Delta_f G^{\circ}_m(\mathrm{g})$, at T = 298.15 K, and the conformational composition, χ_i. 1 a. u. (Hartree) corresponds to 2625.50 kJ·mol-1.

Conformation[1]	$H^{\circ}_{298.15\mathrm{K}}$ [2] / a.u.	$\Delta_f H^{\circ}_m(\mathrm{g})$ [3] / kJ·mol-1	$S^{\circ}_{298.15\mathrm{K}}$ [4] / J·K-1·mol-1	$\Delta_f S^{\circ}_m(\mathrm{g})$ [5] / J·K-1·mol-1	$\Delta_f G^{\circ}_m(\mathrm{g})$ [6] / kJ·mol-1	χ_i [7]
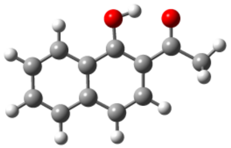	–612.825009	–225.2 ± 3.5	428.25	–499.2	–76.4	1.000
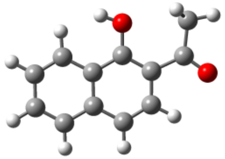	–612.808436	–181.6 ± 3.5	445.95	–481.5	–38.0	0
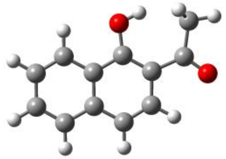	–612.807841	–180.1 ± 3.5	441.61	–485.8	–35.3	0
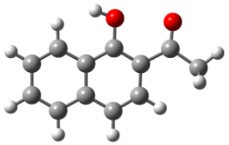	–612.802455	–165.9 ± 3.5	449.25	–478.2	–23.3	0

[1]Spheres color code: grey, C; red, O; white, H.

[2]Obtained from G3(MP2)//B3LYP method [1]

[3]Estimated from 16 working reactions presented on Table 7 of manuscript;

[4]Obtained from B3LYP/6-31G(d) method for a frequency factor scale of 1.0029 [2];

[5]Calculated from $\Delta_f S^{\circ}_m(\mathrm{g}) = S^{\circ}_{298.15\mathrm{K}}$ (conformer i) $- \sum S^{\circ}_{298.15\mathrm{K}}$ (elements), considering the standard absolute entropy elements values, at 298.15 K, $S^{\circ}_{298.15\mathrm{K}}(\mathrm{H_2, g})$ = 130.680 J·K-1·mol-1, $S^{\circ}_{298.15\mathrm{K}}(\mathrm{C, graphite})$ = 5.740 J·K-1·mol-1 and $S^{\circ}_{298.15\mathrm{K}}(\mathrm{O_2, g})$ = 205.147 J·K-1·mol-1 taken from ref. [3];

[6]Calculated from $\Delta_f G^{\circ}_m(\mathrm{g}) = \Delta_f H^{\circ}_m(\mathrm{g}) - T\Delta_f S^{\circ}_m(\mathrm{g})$;

[7]Calculated from $\chi_i = e^{-[\Delta_f G^{\circ}_m(\mathrm{g})/RT]} / \Sigma_i^n e^{-[\Delta_f G^{\circ}_m(\mathrm{g})/RT]}$.

Table S7. Absolute standard enthalpies, $H^\circ_{298.15\ \mathrm{K}}$, and entropies, $S^\circ_{298.15\ \mathrm{K}}$, obtained by G3(MP2)//B3LYP composite method for 2-acetyl-3-naphthol and the corresponding derived gas-phase standard molar enthalpies, $\Delta_f H^\circ_m(g)$, entropies, $\Delta_f S^\circ_m(g)$, and Gibbs energy of formation, $\Delta_f G^\circ_m(g)$, at T = 298.15 K, and the conformational composition, χ_i. 1 a. u. (Hartree) corresponds to 2625.50 kJ·mol^{-1}.

Conformation[1]	$H^\circ_{298.15K}$ [2] / a.u.	$\Delta_f H^\circ_m(g)$ [3] / kJ·mol^{-1}	$S^\circ_{298.15K}$ [4] / J·K^{-1}·mol^{-1}	$\Delta_f S^\circ_m(g)$ [5] / J·K^{-1}·mol^{-1}	$\Delta_f G^\circ_m(g)$ [6] / kJ·mol^{-1}	χ_i [7]
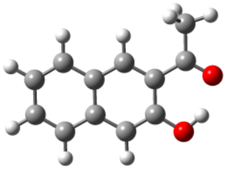	–612.820404	–213.1 ± 3.5	430.68	–496.7	–65.0	1.000
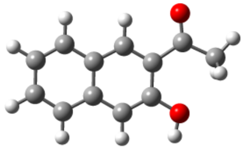	–612.808500	–181.8 ± 3.5	440.93	–486.5	–36.8	0
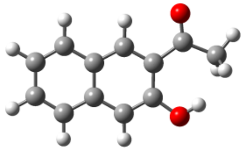	–612.804804	–172.1 ± 3.5	442.47	–485.0	–27.5	0
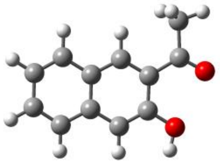	–612.803355	–168.3 ± 3.5	450.36	–477.1	–26.1	0

[1]Spheres color code: grey, C; red, O; white, H.
[2]Obtained from G3(MP2)//B3LYP method [1]
[3]Estimated from 19 working reactions presented on Table 1 of manuscript;
[4]Obtained from B3LYP/6-31G(d) method for a frequency factor scale of 1.0029 [2];
[5]Calculated from $\Delta_f S^\circ_m(g) = S^\circ_{298.15K}$ (conformer i) $- \sum S^\circ_{298.15K}$ (elements), considering the standard absolute entropy elements values, at 298.15 K, $S^\circ_{298.15K}(H_2, g)$ = 130.680 J·K^{-1}·mol^{-1}, $S^\circ_{298.15K}$ (C, graphite) = 5.740 J·K^{-1}·mol^{-1} and $S^\circ_{298.15K}(O_2, g)$ = 205.147 J·K^{-1}·mol^{-1} taken from ref. [3];
[6]Calculated from $\Delta_f G^\circ_m(g) = \Delta_f H^\circ_m(g) - T\Delta_f S^\circ_m(g)$;
[7]Calculated from $\chi_i = e^{-[\Delta_f G^\circ_m(g)/RT]} / \sum_i^n e^{-[\Delta_f G^\circ_m(g)/RT]}$.

Table S8. G3(MP2)//B3LYP enthalpies, $H^{\circ}_{298.15\mathrm{K}}$, and experimental gas-phase standard (p° = 0.1 MPa) molar enthalpies of formation, $\Delta_f H^{\circ}_m$(g), at T = 298.15 K, for *o*-acetylnaphthol isomers and for the auxiliary species. 1 a. u. (Hartree) corresponds to 2625.50 kJ·mol^{-1}.

Compound	Molecular structure	$H^{\circ}_{298.15\mathrm{K}}$ / a. u.	$\Delta_f H^{\circ}_m$(g) / kJ·mol^{-1}
acetophenone	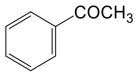	−384.271907	−86.7± 1.7 [4]
1-acetyl-2-naphthol	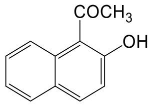	−612.814664	—
2-acetyl-1-naphthol	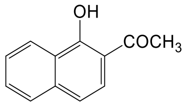	−612.825009	—
2-acetyl-3-naphthol	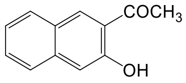	−612.820404	—
benzaldehyde	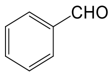	−345.027584	−36.7 ± 2.8 [5]
benzene	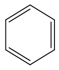	−231.835164	82.6 ± 0.7 [5]
1-formylnaphthalene	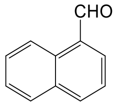	−498.414307	36.3 ± 4.1 [6]
1-hydroxynaphthalene (or 1-naphthol)	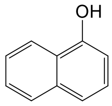	−460.375351	−30.8 ± 1.6 [7]
1-hydroxy-2-naphthaldehyde	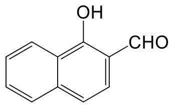	−573.579722	−179.8 ± 7.4 [8]
2′-hydroxyacetophenone	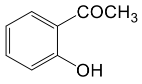	−459.433837	−291.8 ± 2.1 [9]
2-hydroxybenzaldehyde	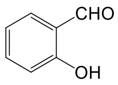	−420.188744	−245.6 ± 2.2 [10]
2-hydroxy-1-naphthaldehyde	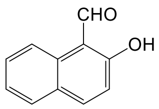	−573.575493	−164.7 ± 2.5 [8]

…/…

Table S8. (Continuation)

Compound	Molecular structure	$H^{\circ}_{298.15\mathrm{K}}$ / a. u.	$\Delta_f H^{\circ}_m$(g) / kJ·mol^{-1}
2-hydroxynaphthalene (or 2-naphthol)	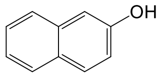	−460.375409	−29.9 ± 1.7 [7]
3-hydroxy-2-naphthaldehyde	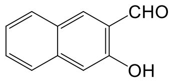	−573.575691	−169.2 ± 7.4 [8]
methoxybenzene	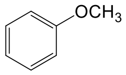	−346.205248	−67.9 ± 0.8 [5]
1-methoxynaphthalene	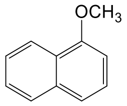	−499.594709	−3.0 ± 3.1 [6]
1-methoxy-2-naphthaldehyde	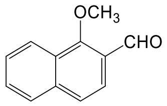	−612.782160	−107.9 ± 3.1 [8]
2-methoxy-1-naphthaldehyde	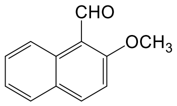	−612.780678	−101.5 ± 2.4 [8]
3-methoxy-2-naphthaldehyde	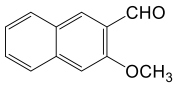	−612.785396	−116.5 ± 3.1 [8]
naphthalene	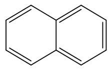	−385.223772	150.3 ± 1.4 [5]
phenol	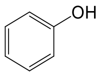	−306.986473	−96.4 ± 0.9 [5]

Table S9. Group substitution reactions for 1-acetyl-2-naphthol, 2-acetyl-1-naphthol, and 2-acetyl-3-naphthol and corresponding calculated values for the enthalpies of formation, $\Delta_f H^{\circ}_m(g)$, in the gaseous-phase, at T = 298.15 K.[1]

Group Substitution Reactions		**1-acetyl-2-naphthol**<br>X=COCH3<br>Y=OH<br>Z=H	**2-acetyl-1-naphthol**<br>X=OH<br>Y=COCH3<br>Z=H	**2-acetyl-3-naphthol**<br>X=H<br>Y=OH<br>Z=COCH3
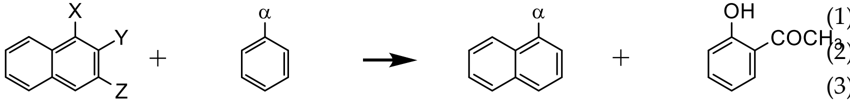	(1) α=H	–203.7	–230.8	–218.7
	(2) α=OCH_3	–204.2	–231.4	–219.3
	(3) α=OH	–205.1	–232.2	–220.1
	(4) α=CHO	–203.3	–230.5	–218.4
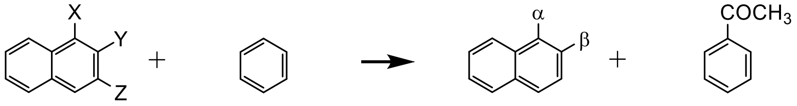	(5) α=OH, β=H	–206.8	–234.0	–221.9
	(6) α=H, β=OH	–205.8	–233.0	–220.9
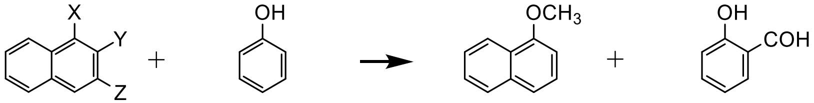	(7)	–198.6	–225.8	–213.7
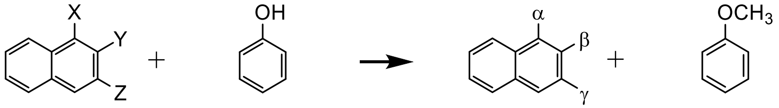	(8) α=CHO, β=OH, γ=H	–189.7	–216.9	–204.8
	(9) α=OH, β=CHO, γ=H	–204.8	–232.0	–219.9
	(10) α=H, β=CHO, γ=OH	–193.7	–220.9	–208.8
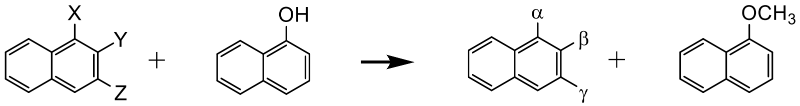	(11) α=CHO, β=OH, γ=H	–188.9	–216.1	–204.0
	(12) α=OH, β=CHO, γ=H	–192.9	–220.1	–208.0
	(13) α=H, β=CHO, γ=OH	–192.9	–200.1	–208.0
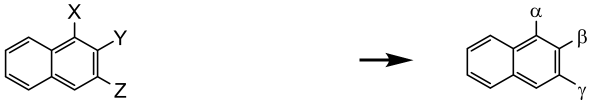	(14) α=OCH_3, β=CHO, γ=H	–193.2	–220.4	–208.3
	(15) α=CHO, β=OCH3, γ=H	–190.7	–217.9	–205.8
	(16) α=H, β=CHO, γ=OCH_3	–193.3	–220.5	–208.4
	Mean value	<–198.0 ± 3.5>[1]	<–225.2 ± 3.5>[1]	<–213.1 ± 3.5>[1]

[1]The quoted uncertainty defines an interval having a 0.95 level of confidence (coverage factor used k = 2.131 for 15 degrees of freedom).

Table S10. Gibbs energies, $G^{\circ}_{298.15\ \mathrm{K}}$, obtained by G3(MP2)//B3LYP composite method for *o*-acetylnaphthol (enol-tautomers) and the matching keto-tautomers, and the theoretically predicted gas-phase standard molar Gibbs energies, $\Delta_r G^{\circ}_m(g)$, for the enol↔keto equilibrium, at T = 298.15 K, with the corresponding fractions (x) of the two tautomers. 1 a. u. (Hartree) corresponds to 2625.50 $\mathrm{kJ{\cdot}mol^{-1}}$.

	enol form	keto form
Molecular structure [1]	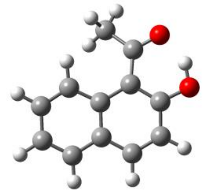 **1-acetyl-2-naphthol**	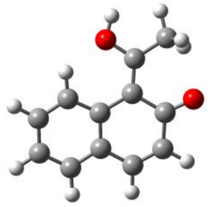 **(*E*)-1-(1-hydroxyethylidene)-naphthalen-2(1*H*)-one**
$G^{\circ}_{298.15\ \mathrm{K}}$ / a. u.	−612.863650	−612.836336
$\Delta_r G^{\circ}_m$(g)[2] / $\mathrm{kJ{\cdot}mol^{-1}}$	71.7	
Fraction[3]	x_{enol} = 1.0	x_{keto} = 0
Molecular structure [1]	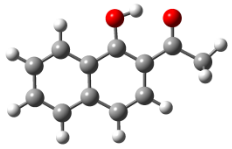 **2-acetyl-1-naphthol**	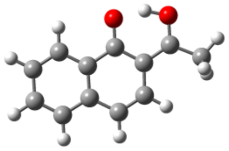 **(Z)-2-(1-hydroxyethylidene)-naphthalen-1(2*H*)-one**
$G^{\circ}_{298.15\ \mathrm{K}}$ / a. u.	−612.874441	−612.865439
$\Delta_r G^{\circ}_m$(g)[2] / $\mathrm{kJ{\cdot}mol^{-1}}$	23.6	
Fraction[3]	x_{enol} = 1.0	x_{keto} = 0
Molecular structure [1]	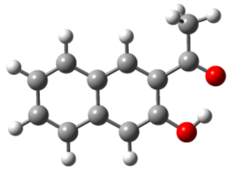 **2-acetyl-3-naphthol**	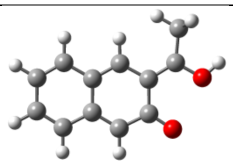 **(Z)-3-(1-hydroxyethylidene)-naphthalen-2(3H)-one**
$G^{\circ}_{298.15\ \mathrm{K}}$ / a. u.	−612.870121	−612.808974
$\Delta_r G^{\circ}_m$(g)[2] / $\mathrm{kJ{\cdot}mol^{-1}}$	160.5	
Fraction[3]	x_{enol} = 1.0	x_{keto} = 0

[1]Most stable conformation; Atom color code: grey, C; red, O; white, H.;

[2] Calculated from $\Delta_r G^{\circ}_m(\mathrm{g}) = G^{\circ}_{298.15\ \mathrm{K}}(\mathrm{keto}) - G^{\circ}_{298.15\ \mathrm{K}}(\mathrm{enol})$;

[3] Calculated from $x_{\mathrm{keto}} = \frac{e^{-[\Delta_r G^{\circ}_m/RT]}}{1+e^{-[\Delta_r G^{\circ}_m/RT]}}$ and $x_{\mathrm{enol}} = 1 - x_{\mathrm{keto}}$.

References

1. Baboul, A. G.; Curtiss, L. A.; Redfern, P. C.; Raghavachari, K. Gaussian-3 theory using density functional geometries and zero-point energies. *J. Chem. Phys.* **1999**, *110*, 7650-7657. https://doi.org/10.1063/1.478676
2. Merrick, P.; Moran, D.; Radom, L. An evaluation of harmonic vibrational frequency scale factor, *J. Phys. Chem. A* **2007**, *111*, 11683-11700. https://doi.org/10.1021/jp073974n
3. Chase Jr., M. W. Nist-Janaf Thermochemical Tables. *J Phys Chem Ref Data* **1998**, Monograph 9 (part I and II), 1-1951. Available online: https://janaf.nist.gov/ (accessed on 20 july 2020).
4. Cox, J. D.; Pilcher, G. *Thermochemistry of Organic and Organometallic Compounds*; Academic Press: New York, 1970.
5. Pedley J. B. *Thermochemical data and structures of organic compounds*. College Station, Thermodynamics Research Centre: Texas, USA, 1994; Volume. 1.
6. Silva, A. L. R.; Freitas, V. L. S.; Ribeiro da Silva, M. D. M. C. Effects of methoxy and formyl substituents on the energetics and reactivity of α-naphthalenes: A calorimetric and computational study. *Chemosphere* **2014**, *107*, 203–210. https://doi.org/10.1016/j.chemosphere.2013.12.044
7. Ribeiro da Silva, M. A. V.; Ribeiro da Silva, M. D. M. C.; Pilcher, G. Enthalpies of combustion of 1-hydroxynaphthalene, 2-hydroxynaphthalene, and 1,2-, 1,3-, 1,4-, and 2,3-dihydroxynaphthalenes. *J. Chem. Thermodyn.* **1988**, *20*, 969-997. https://doi.org/10.1016/0021-9614(88)90225-X
8. Amaral, L. M. P. F.; Freitas, V. L. S.; Gonçalves, J. F. R.; Barbosa, M.; Chickos J. S.; Ribeiro da Silva, M. D. M. C. The influence of the hydroxy and methoxy functional groups on the energetic and structural properties of naphthaldehyde as evaluated by both experimental and computational methods. *Struct. Chem.* **2015**, *26*, 137–149. https://doi.org/10.1007/s11224-014-0475-6
9. Bernardes, C. E. S.; Minas da Piedade, M. E. Energetics of the O−H bond and of intramolecular hydrogen bonding in $HOC_6H_4C(O)Y$ (Y = H, CH_3, $CH_2CH{=}CH_2$, $C{\equiv}CH$, CH_2F, NH_2, $NHCH_3$, NO_2, OH, OCH_3, OCN, CN, F, Cl, SH, and SCH_3) Compounds. *J. Phys. Chem. A* **2008**, 112, 10029–10039**.** https://doi.org/10.1021/jp804455u
10. Ribeiro da Silva, M. D. M. C.; Araújo, N. R. M. Thermochemical studies on salicylaldehyde and salicylamide. *J. Chem. Thermodyn*. **2007**, *39*, 1372-1376. https://doi.org/10.1016/j.jct.2007.03.006
